# Evaluation of airway inflammation in mechanically ventilated patients using cell count and protein concentration

**DOI:** 10.1038/s41598-021-99262-4

**Published:** 2021-10-05

**Authors:** Rodopi Stamatiou, Vasiliki Tsolaki, Apostolia Hatziefthimiou, Epaminondas Zakynthinos, Demosthenes Makris

**Affiliations:** 1 Intensive Care Unit, Medical Department, School of Health Sciences, University Hospital, University of Thessaly, BIOPOLIS, 41500 Larissa, Greece; 2 Laboratory of Physiology, Medical Department, School of Health Sciences, University of Thessaly, BIOPOLIS, Larissa, Greece

**Keywords:** Biological techniques, Biomarkers, Health care, Medical research

## Abstract

Mechanically ventilated (MV) patients may present airway inflammation and elevated secretion production. However, it is unknown whether cell and/or protein counts in bronchial samples may be useful to evaluate their clinical condition. Our aim was to standardize sampling and propose a new mechanical mucus dissolution in Tracheal-Bronchial secretions. In all patients, bronchial lining fluid aspiration (BLF), Bronchoalveolar lavage (BAL) and Bronchial Washings (BW40, BW5) were performed, while visible bronchial secretions were obtained via bronchoscopy (VBS) and blinded, via a common catheter for tracheobronchial aspiration (AC). Mucus was mechanically or DTT dissolved and cell number was count. Protein, albumin and TNF-α levels were measured, in mucus dissolved samples from control and MV patients. Cell number and protein levels were elevated in mucus dissolved compared to non-dissolved, or DTT dissolved. Cell number and TNF-α levels were elevated in MV patients compared to controls, while protein levels were lower in MV patients. Differences in cell and protein levels were observed in samples acquired using different sampling technics. Therefore, mechanical mucus dissolution provides a proper sample for evaluation, and the sampling technic used can influence the sample’s characteristics.

## Introduction

Mechanically ventilated patients may often present elevated secretions and airway inflammation^1,2^. It is not clear whether this is a result of lung complications that may occur during the period of mechanical ventilation such as intensive care unit (ICU) acquired respiratory infections, atelectasis, reduce clearance and subsequent retention of secretions^3,4^ or this is due to the mechanical ventilation (MV) per se. The investigation of this phenomenon can improve the understanding of both the initiation and the progression of effects that are connected with the use of the mechanical ventilator and are highly interacting with both airway inflammation and infection^4–6^.

Various sampling methods and technics are currently available to evaluate airway inflammation^5^. Counting and identification of the cell type and number which are present in bronchial secretions is a valid method that has been used in the investigation of airway inflammation in spontaneous breathing patients^5,6^. However, the application of such methods in MV patients is challenging due to their clinical heterogeneity and the technical difficulties related to airway sampling^7^. Most of the used techniques use induced sputum samples^8,9^ and microbial presence in bronchial sample cultures^10,11^, especially when ventilator associated pneumonia needs to be diagnosed. However, the comparison between different technics, may reveal some advantages in answering specific clinical questions^10,11^, but not others, for example ventilator induced airway inflammation, and are sometimes difficult to perform in specific MV patients. In addition, samples from MV patients are usually enriched with mucus that represents a challenge in sample handling. Mucus makes the sample hard to handle since it prevents the sample from aliquoting, pipetting and/or smearing and staining. Mucus forms cell clusters in the sample and therefore, needs to be removed in order to evaluate parameters like cell counts, protein markers on cells or protein concentration in the sample. The technics used for mucus dissolution often include chemicals like dithiothreitol (DTT)^8,9^ that can alter the protein background in the sample. Other technics elaborate multiple washings^12^, needle aspiration^13^ or enzymic incubation^14^, that can affect the characteristics of the sample.

In this prospective study, we aimed to standardize a method of airway sampling and processing in MV patients by sampling different bronchial sites, using bronchoscopy. This technic might be efficient in evaluating a number of parameters that can be helpful for clinical and research purposes.

### Patients-samples

This was an observational prospective case–control study contacted in the University Hospital of Larissa, Greece between 2019 and 2020. All patients and/or patients’ next of kin were informed of the study and they provided the informed consent. The study was approved by the ethics committee of the Medical Department and University Hospital, School of Health Sciences, University of Thessaly, adhered to the Helsinki Declaration and was reported on the basis of the STROBE guidelines.

Cases were recruited by consecutive sampling among ICU patients if they had undergone bronchoscopy within 24 h from ICU admission and were expected to be mechanically ventilated for at least 10 days. Controls were recruited if they required to undergo planned bronchoscopy for diagnostic purposes (solitary lung nodules). Exclusion criteria were for both controls and patients a) any type of inflammatory airway disease, b) inhaled antibiotic treatment, c) corticosteroids, d) acute cardiogenic pulmonary edema, e) active Tuberculosis, f) hemoptysis.

Samples were acquired at the first 24 h of intubation, with samples being acquired from 35 MV patients and 5 controls. Six different consecutive samples were obtained: a) by blind bronchial aspiration with a common aspirating tube by the attending nurse before bronchoscopy (AC), and by aspirating via bronchoscopy b) macroscopically visible secretions (if any) from the main bronchi (VBS), c) the surface of areas with no macroscopically visible secretions on one main bronchus (BLF), d) the surface of areas with no visible secretions on the other main bronchus after the instillation of 5 ml NaCl 0.9% (BW5), e) segmental airways after the instillation of 40 ml NaCl 0.9% (BW40) f) segmental airways after the instillation of 120 ml NaCl 0.9% (BAL). In control subjects the samples were acquired only by bronchoscopy. In the experiments where TNF-α levels were evaluated, samples from 5 control and 14 MV patients were used.

### Sample handling

Each sample was divided in three sub-samples, where only in one of them mucus was mechanically dissolved using a Heidolph Silent Crusher S (Heidolph Instruments GmbH & Co. KG, Germany), the other one was used without any dissolution and in the third one the mucus was dissolved using a 15 min incubation with 0.2% DTT^8^. In all sub-samples microscopical observation and cell count was performed after Trypan blue staining. Furthermore, total protein, as well as albumin concentration, was estimated in samples with or without cells using the Bradford method and the Bromocresol green chromatographic method, respectively.

TNF-α levels were evaluated in mucus dissolved samples from controls and from 14 MV patients, using the Human TNF-α ELISA ^PRO^ KIT (MABTECH) according to the manufacturer’s instructions.

### Statistical analysis

All data were expressed as means ± SEM and N refers to the number of independent samples. Differences between means were analyzed by Wilcoxon, Mann–Whitney or ANOVA, with statistically significant differences between groups being determined by Bonferonni’s post hoc test. The linear association between cell number or protein in mucus with the severity of the disease (Apache II and SOFA Score), blood white cell number and C-reactive protein plasma level was done with Spearman correlation. A comparison was considered significant when *p* < 0.05. The statistical analysis was performed using Graph Prism.

## Results

Thirty-five MV patients and five control subjects were included in the study, with a mean age of 65.9 ± 3.25 years. The MV patients, in the day of admission, had a mean Apache II score of 18.33 ± 1.93 and a mean SOFA (Sequential Organ Failure Assessment) score of 8.81 ± 0.93. Even more, C-reactive protein (CRP) plasma level was 11.05 ± 1.85 mg/L, white cell number (WBC) in blood was 14,983 ± 1555 and plasma albumin was 2.88 ± 0.17 mg/L.

**Figure 1:**
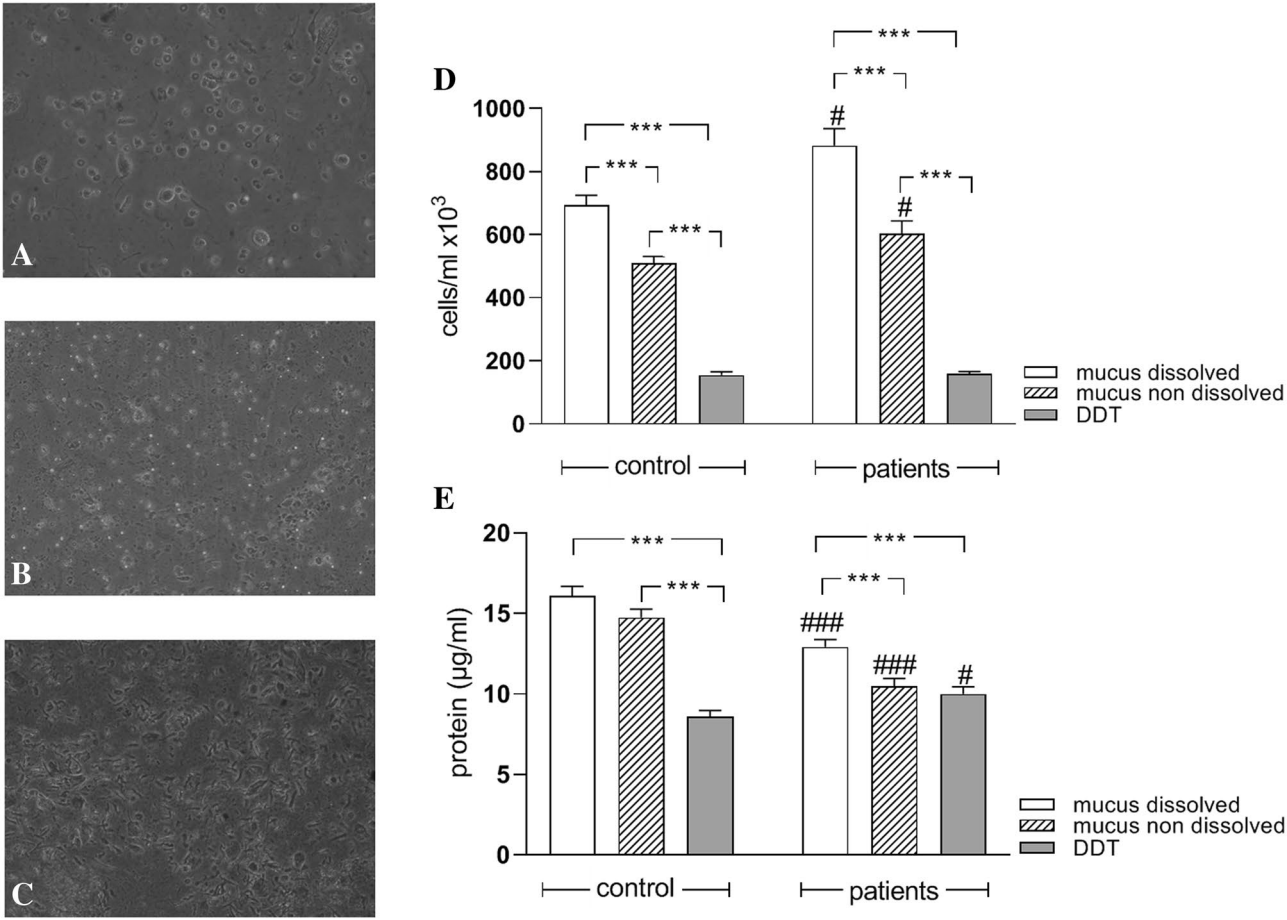
Images of mucus dissolved (**A**), mucus non dissolved (**B**) and dithiothreitol (DDT) dissolved (**C**) samples at 20 × magnification. Cell counts (**D**) and protein levels (**E**) in samples from control and mechanically ventilated patients, with mechanical dissolution (mucus dissolved), without mechanical dissolution (mucus non dissolved) or DDT procedure. Values are presented as mean ± SEM of 25 samples from control and 48 samples from mechanically ventilated patients. ***p < 0.001 for comparisons between groups (ANOVA Bonferonni’s post hoc test) and #*p* < 0.05, ###*p* < 0.001 compared to the relative control samples (Mann–Whitney test).

Samples received from five control subjects and eight MV patients via AC, BLF, BAL, bronchial washings (BW5 and BW40) or VBS were processed in parallel with mechanical mucus dissolution or DDT dissolution. The mucus dissolution allowed an easy sample handling such as pipetting, aliquoting, and dividing into sub-samples. Furthermore, the fact that cells were not attached in clusters of mucus (Fig. 1A), in mucus-dissolved samples, as observed in non-dissolved samples (Fig. 1B), enhanced cell count (Fig. 1D) and made cell handling, such as smearing and staining, both fast and smooth. Thus, cell number counts were significantly (p < 0.01) elevated in mucus dissolved samples compared to non-dissolved ones taken from either control subjects or mechanically ventilated patients (Fig. 1D). The mechanical dissolution of mucus did not affect protein levels in control subjects (Fig. 1E) but increased significantly protein levels in MV patients. Samples proceeded with DDT (Fig. 1C) had significantly decreased cell number and protein levels in either control or MV patients compared to mucus-dissolved samples (Fig. 1D). Even more, when mucus was mechanically dissolved, the samples taken from MV patients showed significant increase in cell number (Fig. 1D) and a decrease in protein levels (Fig. 1E) compared to control. These differences were not noticed when samples were proceeded with DDT and in fact protein levels were increased in DDT—proceeded samples from MV patients compared to control. The differences described above were observed in all sampling technics (data not shown).

**Figure 2:**
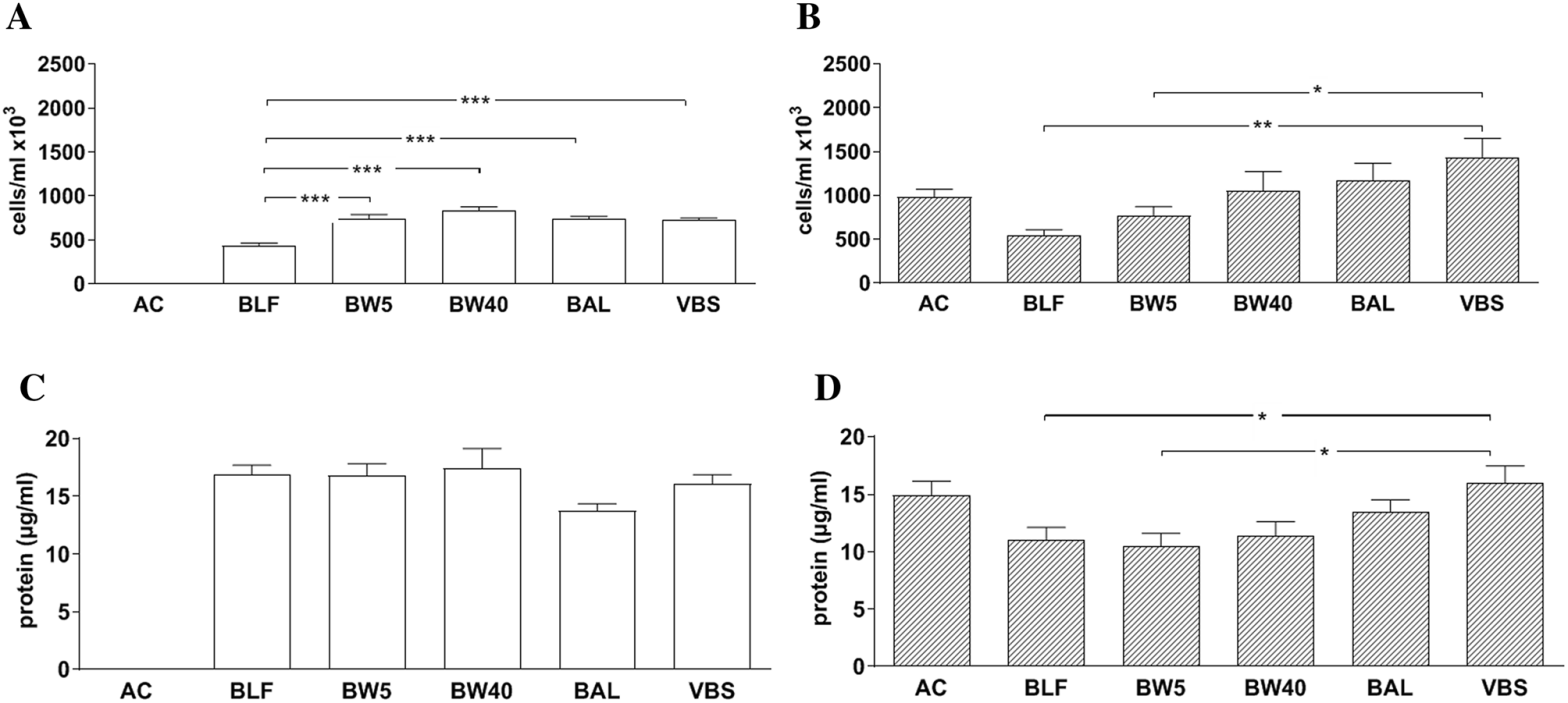
The effect of sampling technic on cell number (upper panel) and protein concentration (lower panel) in mucus dissolved samples obtained from five control subjects (**A**,**C**) or thirty-five mechanically ventilated patients (**B**,**D**). Sampling was performed via tracheobronchial aspiration (AC), aspiration of bronchial lining fluid (BLF), bronchoalveolar lavage (BAL), bronchial washings (BW40 and BW5) or visible bronchial secretions obtained via bronchoscopy (VBS). Data are presented as mean ± SEM of independent samples with *p < 0.05, **p < 0.01 and ***p < 0.001 for comparisons between sampling methods (ANOVA Bonferonni’s post hoc test).

Then we evaluated the effect of sampling technic on cell number and protein concentration in samples collected from control subjects and MV patients. The mechanical mucus dissolution was chosen for processing the samples and protein levels were measured without cell removal. These experiments showed that the sampling technic mainly affected the number of cells and in a smaller degree the protein levels of the samples (Fig. 2). In particular, in control subjects, cell number was significantly (p < 0.001) decreased in BLF samples compared to the cell number of samples obtained with all other technics (Fig. 2A). In MV patients, cell number was significantly increased (p < 0.05) in VBS samples compared to BW5 or BLF samples (Fig. 2B). On the other hand, the sampling technic had no effect on protein levels in control subjects (Fig. 2C) while in MV patients significant differences (p < 0.05) in protein levels were observed only between samples taken via VBS and BW5 or BFL (Fig. 2D). Whatever technic was used to collect the sample, no correlation was found between cell number and protein levels.

Furthermore, we measured TNF-α levels in mucus samples of 14 MV patients obtained via differential sampling technics and no differences in values were found (Table 1). Significant differences in cell number, protein and TNF-α levels in dissolved mucus, between controls and MV patients depended on the sampling technic (Table 1). Specifically, cell number was increased in patients compared to controls when samples obtained via VBS, protein levels was decreased in patients compared to controls when samples obtained via BLF or bronchial washings (BW5, BW40) and TNF-α levels were increased in patients compared to controls when samples obtained via bronchial washings or BAL (Table 1).

**Table 1:** Cell number, protein and TNF-α levels, in mucus dissolved samples obtained control subjects and mechanically ventilated (MV) patients.

	Sampling technic					
	AC	BLF	BW5	BW40	BAL	VBS
**Cells/ml × 10**^**3**^						
Control (N = 5)	–	438 ± 24	745 ± 43	830 ± 46	735 ± 32	726 ± 21
MV patients (N = 35)	978 ± 92	544 ± 64	772 ± 99	1052 ± 218	1173 ± 193	1431 ± 216*
**Protein levels (μg/ml)**						
Control (N = 5)	–	16.9 ± 0.8	16.8 ± 1.0	17.4 ± 1.7	13.8 ± 0.6	16.1 ± 0.8
MV patients (N = 35)	14.9 ± 1.2	11.0 ± 1.1*	10.5 ± 1.1*	11.4 ± 1.3*	13.5 ± 1.0	16.0 ± 1.5
**TNF-α (pg/ml)**						
Control (N = 5)	–	405 ± 152	365 ± 110	487 ± 88	563 ± 59	678 ± 153
MV Patients (N = 14)	953 ± 112	837 ± 147	785 ± 86*	859 ± 111*	985 ± 119*	946 ± 135

Sampling with tracheobronchial aspiration (AC), aspiration of bronchial lining fluid (BLF), bronchoalveolar lavage (BAL), bronchial washings (BW5, BW40) and visible bronchial secretions obtained via bronchoscopy (VBS). N refers to the number of subjects and data are presented as means ± SEM. *p < 0.05 for comparisons between control and patients (Mann–Whitney test).

In the thirty-five MV patients we evaluated the correlation between cell number and protein levels in mucus with the severity of the disease (Apache II and SOFA Score), WBC number and CRP or plasma albumin levels. We found no significant correlation between sample protein level or cell number with Apache II, SOFA Score, WBC, CRP and albumin levels.

## Discussion

In the presented method the mucus in bronchial aspirates was being mechanically dissolved, providing an easy to use sample, that can be used for cell and protein evaluation. The presence of mucus, represents a challenge in handling bronchial samples and makes the mucus dissolution necessary. There are various mucus dissolving technics, such as needle aspiration^13^, repeated washing^12^ or enzyme use^15^. However, all these technics are not easy to perform and leave a part of the sample inappropriate to use, reducing the sample volume that can be used for various evaluations.

The most commonly used technic for bronchial sample handling is the use of dithiothreitol (DTT), as a mucus dissolving agent. DTT is used in order to reduce the disulfide bonds on mucus glycoproteins, and therefore release cells and proteins trapped in mucus clusters. After the chemical dissolution the sample is filtrated^8,9^. DTT is used for mucus dissolution in sputum samples where cell number is count or evaluation of various protein levels^8,9^. However, the protocols used reduce both the number of cells in the sample and the protein concentration. Furthermore, the addition of DTT does not dissolve all mucus clusters. DTT has been found to affect leukocyte surface markers essential for leukocyte subtyping^16^, as well as the presence and concentration of proteins^17^. On the contrary, the mechanical mucus dissolution in the present study completely eliminated mucus clusters compared to the non-dissolved samples or the DTT dissolved samples, showing that the mechanical mucus dissolution allows us to have a sample easier and more appropriate to use for testing. This technic did not negatively affect cell count or chemical context of samples but on the contrary allowed higher cell numbers to be count and protein concentration, respectively, indicating that mechanical dissolution of mucus could represent a new and more efficient handling technic.

Sampling technique seemed to affect either cell counts or protein concentration. However, the fact that cell number and protein levels in AC samples does not significantly compared to those in samples acquired by bronchoscopic methods, is interesting. These data could suggest, that AC could be an easy and probably effective technique for sampling in MV patients. Data presented in previous studies show that endotracheal aspirates can be used instead of BAL, for example, in the diagnosis of VAP^11^.

The assessment of clinically relevant readouts of the method was not the main object of this study. However, as only MV patients without lung disease were included in the study, the fact that no correlation was found between disease severity (estimated with Apache II and SOFA score) and inflammation (estimated with WBC and CRP) suggests that the presence of an underlying disease without lung infection does not affect sample cell number and protein levels. Subject to the small number of MV patients in whom it TNFA-a was measured in mucus, there are differences between controls and MV patients that concern mainly protein and TNF-a levels in mucus samples.

The presence of TNF-a in the BW5, BW40 and BAL samples in MV patients could be an indication of inflammation, since this cytokine is usually used for inflammation evaluation^5,6^. The fact that TNF-a could be effectively measured in mucus dissolved samples enhances the diagnostic value of this method, since it proves that it can be used for inflammation evaluation, through cytokine’s measurement in MV patient samples.

In conclusion, the technic used in this study introduced two main innovations. Firstly, the comparison between different sample acquisition technics that could be used revealed differences that can be of use in sample acquisition in order to evaluate inflammation in MV patients. Furthermore, the mechanical mucus dissolution allowed a fast and easy way of sample handling that could not only provide proper samples for lab testing but also further examination procedures that could not be performed with the same effectiveness, when mucus dissolution was performed chemically, due to DTT effects. Both the sampling and the handling technics that were described in the present study can help clinicians improve bronchial sampling and may provide further insight in the investigation of airway inflammation pathophysiology during MV.
